# Effects of incidental physical activity on morphosyntactic processing in aging

**DOI:** 10.1371/journal.pone.0239727

**Published:** 2020-09-29

**Authors:** Graciela C. Alatorre-Cruz, Javier Sanchez-Lopez, Juan Silva-Pereyra, Thalía Fernández

**Affiliations:** 1 Facultad de Estudios Superiores Iztacala, Universidad Nacional Autónoma de México, Estado de México, México; 2 Departamento de Neurobiología Conductual y Cognitiva, Instituto de Neurobiología, Universidad Nacional Autónoma de México, Querétaro, México; 3 Department of Neurosciences, Biomedicine and Movement Sciences, University of Verona, Verona, Italy; 4 Centro de Investigación en Ciencias Cognitivas, Universidad Autónoma del Estado de Morelos, Morelos, México; University of Central Florida, UNITED STATES

## Abstract

Older adults have difficulties in sentence comprehension when working memory (WM) load increases (e.g., multiple embedded clauses). Structured physical activity has been related to improvements in cognition; however, incidental physical activity (PA, i.e., unstructured daily physical activities), particularly incidental vigorous activity has been poorly studied in relation to its effects on behavior. Furthermore, no positive effect on language has been reported in either form of physical activity. The aim of this study was to evaluate how two levels of PA (high or low) affect WM processing and how this, in turn, may affect morphosyntactic processing in older adults. Individuals with high PA (n = 18) had a higher WM load effect than those with low PA (n = 18), both behaviorally (greater differences between high and low WM loads in correct responses) and in terms of event-related potentials (only subjects with high PA showed LAN and P600b amplitude differences between high and low WM loads). These findings suggest that PA promotes cognitive strategies to face WM loads and morphosyntactic processing.

## Introduction

Aging entails cognitive changes that, in older adults, can manifest as a decline in sustained attention, selective inhibition [1], selective attention [2, 3], divided attention [3, 4], perception [2], and episodic memory [5]. However, the most acute changes have been observed in processing speed [6] and working memory (WM; [6, 7]).

Although language is regarded as a crystallized cognitive process [6], important changes in sentence processing have been reported in older people, particularly in the comprehension of embedded syntactic structures [8, 9] and in morphosyntactic processing [10, 11]; this latter process involves agreement rules between lexical units (e.g., in the phrase “He runs,” the pronoun “He” inherits the number agreement to the verb “run” and the suffix “s” is added). The features used to compute agreement are the marking of gender, number, person or case [12]. It has been suggested that a larger number of lexical units make language comprehension more difficult because subjects have to maintain grammatical information in WM [13]. Therefore, we postulate that morphosyntactic processing may also be affected by an age-related decline in WM processing.

Several studies have suggested that lifestyle also affects cognition in aging (see [14] for a systematic review and [15, 16] for two of the most recent studies). Specifically, physical activity seems to positively influence cognitive functions [17, 18] due to variations in hematological [19] and hormonal parameters [20] produced by changes in the circulatory, respiratory and muscular systems [21].

There are two main approaches to studying the associations between physical activity and improved cognition in aging related to the level physical activity planning (i.e., the level of a deliberate selection of the type of activity and its frequency, volume and intensity): structured physical activity (either short or long term), which is planned and repetitive and is used to enhance one or more components of physical fitness to improve physical skills, fitness or health [17, 22], and incidental physical activity (PA), which is considered the result of unstructured daily activities, such as housekeeping, working, transportation, etc. [23]. It is important to note that the above-mentioned distinction refers to the level of structure in the planification of the activity, however both types of physical activity could be performed at different levels of intensity, i.e. moderate to vigorous, which depends of the energy expenditure related to the activity. The measurement of PA requires the use of instruments able to discriminate between structured and unstructured physical activities. The self-report inventory, where the participant is asked for his/her involvement in different activities of the diverse domains of daily life, is a good way to assess and distinguish the level of PA from that of structured activities. Different instruments have been developed to evaluate PA in older adults; in our study, we adopted the Yale Physical Activity Survey (YPAS, Spanish version [24]; see below for a detailed description), which has been shown to be suitable for the evaluation of PA in older adults and has demonstrated high reliability and correlation with accelerometry parameters (see, for example, [25]).

Several previous studies have suggested that structured physical activity positively influences WM processes and executive function [26–29], and it has also been reported that structured physical activity and PA generate similar effects on physiological and cognitive processes [30, 31]; therefore, we hypothesize that PA may also improve WM process, which in turn may affect morphosyntactic processing because this process requires the maintenance of available information to be efficient. However, although sentence processing has been studied previously (see Etnier et al. for a meta-analysis [32] where there were no conclusive results for the association between physical exercise and sentence processing), to our knowledge, no studies have been reported regarding the effect of structured or unstructured physical activity on morphosyntactic processing. Therefore, we propose to assess the effect of PA on morphosyntactic processing at two different WM load levels. To study this hypothesis, event-related potentials (ERPs) were recorded. ERP studies can provide valuable information about brain activity concurrent with morphosyntactic processing or other cognitive processes; that is, ERPs may help to tease out the influence of PA. ERPs are the averages of brain electrical activities that are time-locked to external or internal stimuli [33]. For instance, if a person reads a well-formed sentence while his/her ERPs are recorded, a negative wave is observed over the left anterior sites of the scalp. However, when the person reads a syntactically anomalous sentence, this same wave will have a greater amplitude because the anomaly requires additional computation; the amplitude difference between a well-formed sentence and anomalous sentence is called the effect.

In language studies of gender agreement, a negative wave occurs between 300 and 500 ms after stimulus onset and peaks at approximately 400 ms, known as the left anterior negativity (LAN) component, and its amplitude modulation depends on morphosyntactic violation detection, that is, the identification and matching of morphosyntactic partners word by word [34–37].

Particularly, in morphosyntactic processing, gender agreement has been amply studied in different languages using ERPs in young and older subjects, and this agreement manipulation seems to be concurrent with a pattern of ERP components [33]. When subjects process sentences in Spanish, the LAN-P600 waveform pattern has been commonly observed [8, 34, 35]. In young participants, a larger LAN amplitude (but smaller LAN effect; the disagreement condition minus the agreement condition) has been reported due to WM load demands (i.e., the complexity or length of the sentence, [38]). A smaller P600 effect and longer P600 latencies have been found when morphosyntactic complexity was increased [38–40]; both LAN and P600 modulation were associated with longer reaction times and poorer sentence comprehension [38]. According to some authors, the P600 component might reflect two consecutive processing steps [41–43]. The first step (reflected by P600a, found between 500 and 700 ms) integrates all of the information associated with the previous sentence context [41, 44, 45]. In the second step (reflected by P600b, found between 700–900 ms), a generalized mapping of sentences [34] may be performed [34, 41, 44].

However, as people get older, language processing becomes associated with longer latencies and smaller amplitudes of many ERP components [11]. There are few studies analyzing ERP age-related changes during sentence comprehension when either grammatical agreement or WM load was manipulated. For instance, a study showed a more asymmetric and frontal topographic distribution of the P600 in older adults than in young participants when number agreement was manipulated [46], while only one study performed in aging adults reported the effect of high WM load in morphosyntactic processing using gender-agreement manipulation [10]. The authors described that healthy older adults showed a smaller LAN effect than young participants as well as smaller amplitudes in P600a and P600b effects in high WM load condition compared with those in low WM load condition, a finding that was not observed in young participants [10]. These findings were interpreted as a greater neural cost to compute morphosyntactic processing as well as to integrate the information associated with the previous sentence context and to generate a generalized sentence mapping. The more complex condition, in this case, is the disagreement condition due to an additional computation of gender manipulation; however, older adults showed similar accuracy to young participants regardless of WM load; therefore, the electrophysiological pattern associated with the high WM load condition could be considered a compensatory response [47].

Therefore, considering that PA and structured physical activity produce similar physiological changes and that both induce cognitive improvements in aging, we hypothesize that PA could influence language processing by positive changes in WM. This study aimed to assess sentence comprehension in two WM load conditions in two groups of older adults: one with a high level of PA (h-PA) and the other with a low level of PA (l-PA). We expected that older adults with l-PA would show longer reaction times (RTs) and have fewer correct answers than older adults with h-PA and that these differences between groups would be more evident in the high WM load condition. Given that a higher WM load imposes a greater cost associated with agreement processing, we expected that older adults with l-PA would show greater difficulties computing morphosyntactic processing, which may be reflected as a smaller LAN effect, and problems integrating all information with the previous sentence context (i.e., smaller P600a and P600b effects) than the group with h-PA as WM load is increased.

## Method

### Participants

A previously studied database of 97 older adults aged 60 years and older (mean age = 66.80 years, SD = 4.30), recruited using nonprobabilistic sampling by convenience, was used to select the participants of the present study. According to a previous study [31], this database was divided into two groups (h-PA and l-PA) by a cluster analysis using the Ward method with a measure of squared Euclidean distance. Such statistical analysis took into account the total kcal/week, vigorous activity index, and moving index, which are variables of the YPAS [24]. The YPAS is used to measure the level of physical activity in adults aged 60 and older. The questionnaire is a self-report inventory and is divided into two parts. First, participants are asked about the time spent on housework, working, yard work, caretaking and leisure activities. And in the second part of the questionnaire, the frequency and time spent in vigorous activities, leisurely walking, standing, moving and sitting are asked. Once the participant answers the questionnaire, the evaluator processes the information by filling the score sheets included in the same questionnaire. For the first part, values for time in minutes/week, energy expenditure in metabolic equivalent of task (MET) by time and energy expenditure in kilocalories (MET*time*weight) are obtained for each specific activity. Values for each domain and total value across domains are calculated, as well. In the second part, partial indices are calculated for each activity by multiplying the frequency score by the duration for each activity and multiplying again by a weighting factor, which is based on the relative intensity of the physical activity dimension and provided in the same questionnaire. The sum of the partial indices results in the total index.

The inclusion criteria of the sample were as follows: at least 9 years of schooling and a score of more than 90 on the Spanish version of the Wechsler Adult Intelligence Scale (WAIS, [48]). None of the participants had major socioeconomic disadvantages (evaluated with Regla AMAI NSE 8x7 [49]), evidence of depression, or differences in lifestyle (assessed with a survey of habits about training/information (participating in workshops, learning and using another language, using new technologies, watching, listening to or reading the news); hobbies (participating in activities such as reading, writing, listening or playing music; traveling, attending cultural and artistic events, etc.); aspects of social life (participating in meetings, social, cultural or religious activities). They had normal scores on the brief neuropsychological test battery in Spanish (NEUROPSI [50]), which assesses a wide spectrum of cognitive functions, including orientation attention, memory, language, visuoperceptual abilities and executive functions. All subjects were assessed by a specialist in the area of geriatric psychiatry to exclude participants with any psychiatric or neurological disorder. All participants had to obtain scores of 1 or 2 on the Global Deterioration Scale (GDS, [51]) and a normal score on the Mini-Mental State Examination test (MMSE, [52]), indicating that they did not show behavioral evidence of cognitive decline. Additionally, they were required to have normal scores on the Geriatric Depression Scale [53]. Participants did not present any signs of anemia, diabetes, hypercholesterolemia, or thyroid disease in clinical blood analysis or uncontrolled hypertensive disease.

A sample size calculation from the original database performed to obtain the representative number of participants required for our study determined a total of 78 individuals. Therefore, 78 people were invited to participate in our study, but only 68 (34 in the h-PA group and 34 in the l-PA group) agreed to participate.

All the recruited participants performed a sentence comprehension reading task while their ERPs were recorded. Ultimately, only participants with above-chance performance (> 58.1% of correct response) in the overall task and with no less than 80% of artifact-free EEG segments associated with correct responses, which corresponds to at least 18 segments by condition considering the minimum of corrects responses recorded, were included in the final analysis. The average number of correct responses of the sample was 75.3% (SD = 1.2), while the average number of clean EEG segments across conditions was 30 (SD = 0.5). Finally, 18 individuals with h-PA and 18 individuals with l-PA reached the criteria (see Table 1). Participants included in the final sample were interrogated about their participation in structured physical activities, and seven of them (three in the l-PA group and four in the h-PA) participated in recreational sports (tennis, running and swimming). A two-way chi squared test was performed to evaluate the association between group and recreational sport participation. The results were not significant (Pearson χ^2^ = .17; p = 0.67), suggesting no differences in the frequency of individuals practicing recreational sports between groups.

**Table 1:** Means and Standard Deviations (SDs) of demographic data, socioeconomic status, survey habits, GDS Yesavage, MMSE, NEUROPSI scores, GDS Reisberg, WAIS results, Yale Physical Activity Survey (YPAS) scores, Body Mass Indexes (BMIs) and blood analysis results of the samples.

	h-PA	l-PA	Cohen’s d
	mean (SD)	mean (SD)	(p < .05)
Age	67.00 (4.01) 60–77 y.o.	65.56 (4.06) 60–75 y.o.	
Years of schooling	17.27 (5.02)	15.53 (4.46)	
Regla AMAI^1^	212.22 (28.01)	219.94 (28.50)	
Survey of habits			
Training/Information	11.10 (2.75)	11.64 (2.70)	
Hobbies	30.31 (8.50)	32.50 (7.22)	
Social life	7.74 (2.74)	7.75 (2.50)	
GDS Yesavage	1.18 (.27)	1.99 (.47)	
MMSE*	29.27 (.82)	28.38 (.77)	1.11
NEUROPSI	110.72 (7.43)	110.56 (8.06)	
GDS Reisberg	1 = 17; 2 = 1	1 = 17; 2 = 1	
WAIS IQs			
Verbal IQ	113.38 (8.28)	111.77 (6.80)	
Performance IQ	108.83 (10.13)	102.61 (10.27)	
WAIS indices			
VCI	124.66 (10.30)	122.38 (8.85)	
POI	107.16 (14.30)	109.55 (12.52)	
WMI	105.27 (5.24)	104.11 (4.71)	
PSI	112.44 (19.00)	111.00 (14.90)	
YPAS kcal/week			
Housework	3950.83 (2837.95)	4670.87 (3138.63)	
Work	81.98 (291.09)	910.35 (3347.53)	
Yardwork	486.02 (496.58)	471.68 (545.28)	
Caretaking	1914.03 (3384.51)	1180.97 (1876.35)	
Leisure	3050.18 (1419.19)	2430.82 (1510.77)	
YPAS index			
Vigorous activity***	45.00 (14.14)	10.83 (11.27)	2.67
Leisure walking	11.33 (8.26)	10.00 (6.97)	
Moving	11.33 (3.34)	9.83 (4.46)	
Standing	3.44 (3.34)	3.88 (2.86)	
Sitting	3.05 (1.16)	2.77 (1.16)	
BMI*	23.5 (.48)	25.4 (.67)	3.26
Blood analysis			
Total Cholesterol	207.05 (31.56)	193.76 (30.18)	
Hemoglobin	14.70 (1.16)	14.82 (1.24)	
Glucose	95.43 (12.01)	98.17 (11.94)	
Thyroid-stimulating hormone	2.36 (1.28)	2.09 (1.40)	

*Note*: *significant differences between groups;*

***p < .*001*

*p < .*05;*

^*1*^*socioeconomic status; GDS Yesavage*: *Geriatric Depression Scale Yesavage; MMSE*: *Mini-Mental State Examination; NEUROPSI*: *brief neuropsychological test battery in Spanish; GDS Reisberg*: *Global Deterioration Scale Reisberg*, *frequency of participants for each scores between 1 and 2 in each group are displayed; WAIS*: *Weschler Adult Intelligence Scale; IQ*: *intelligence quotient; VCI*: *verbal comprehension index; POI*: *perceptual organization index; WMI*: *working memory index; PSI*: *processing speed index; YPAS*: *Yale Physical Activity Survey; BMI*: *body mass index*. *Training/information (participating in workshops*, *learning and using another language*, *using new technologies*, *watching*, *listening to or reading the news); hobbies (participating in activities such as reading*, *writing*, *listening or playing music; traveling*, *attending cultural and artistic events*, *etc*.*); social life (participating in meetings*, *social*, *cultural or religious activities)*.

The Ethical Committee of the Institute of Neurobiology at the National Autonomous University of Mexico approved this study. The main incentive for volunteers was that free access to their clinical screening results was provided. All volunteers signed informed consent forms. The entire study was conducted in the Psychophysiology Laboratory of the Institute of Neurobiology at the Universidad Nacional Autónoma de México, Juriquilla, Querétaro, México.

Table 1 shows the demographic information and the WAIS and YPAS scores of the h-PA and l-PA groups. Only the vigorous index showed a significant difference between groups. Members of the h-PA group displayed a more vigorous physical activity index (i.e., participation in high-intensity physical activities lasting at least 10 minutes that cause large increases in breathing, heart rate, leg fatigue or perspiration) and lower BMI than those of the l-PA group.

### Stimuli

Two hundred and twenty sentences previously used elsewhere [10] were used for this study. Stimuli consisted of 160 gender-agreement and -disagreement sentences between the noun of the main clause and its adjective, which were divided into two levels (low and high) of WM load (i.e., syntactic complexity: number of nodes parsed; [13]). Disagreement sentences were built by changing the derivational morpheme indicating the gender of the qualifying adjective, i.e., *rojo–roja* (red _Masculine_−red _Feminine_; in Spanish, the last morpheme indicates masculinity or femininity).

**Fig 1:**
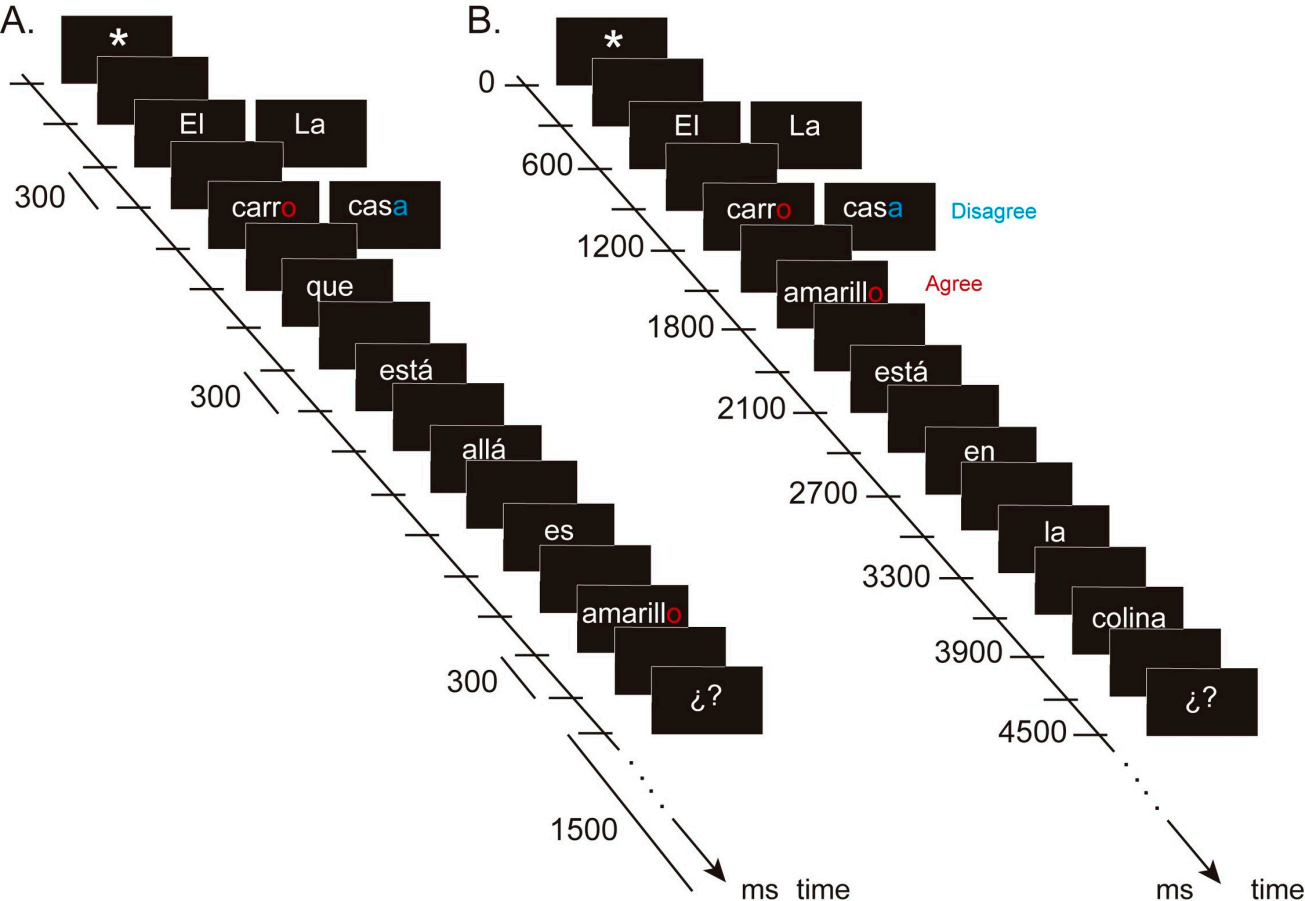
Examples of the comprehension reading task. A) Sentence with high working memory load: “The car [masculine] over there is yellow [masculine or feminine]”. B) Sentence with low working memory load: “The yellow [feminine or masculine] car [masculine] is on the hill”. Red font in the last letter in the noun and the adjective, represent the agree condition; blue font in the last letter of the noun and red font in the last letter of the adjective, represent the disagree condition.

In summary, 40 agreement and 40 disagreement sentences were low WM load sentences, and 40 agreement and 40 disagreement sentences were high WM load sentences in which a clause was embedded within noun-adjective agreement or disagreement. This clause was placed between lexical units with a dependent syntactic relationship (for an example, see Fig 1). Sixty additional sentences were included as fillers, with 30 grammatical and 30 ungrammatical sentences. These sentences had the same syntactic structure but different morphosyntactic manipulations (i.e., number agreement).

The adjective expressed a characteristic of the main noun in the sentence, and all of the nouns designated inanimate objects (with the same proportion of genders).

### Sentence comprehension reading task

The task was presented to subjects using STIM2 software (NeuroScan, CompuMedics, Charlotte, NC, United States) on a computer screen while subjects were seated at a distance of 70 cm from the screen. The words were displayed in white at the center of a black screen, the type font was Arial, and the font size was 80 pt.

Sentences were presented one word at a time for 300 ms each with an interstimulus interval of 300 ms. At the beginning of every sentence, a fixation cross was presented for 300 ms, and at the end of the sentence, two question marks appeared for 1500 ms. Subjects were instructed to read the whole sentence and to respond as efficiently and quickly as possible only when the question marks appeared. They were required to answer whether the sentence was correct (grammatical) or not by pressing one of two buttons using their thumbs on a response box. One button was for “correct” sentences (gender/number agreement), and the other was for “incorrect” sentences (gender/number disagreement). Response buttons were counterbalanced among subjects, such that the correct button for one subject might be the incorrect button for the other subject. The task took 35 minutes; the subjects had three rest periods, one every 9 minutes. It is important to highlight that the task was adjusted to keep a reasonable experimental time to avoid fatigue in older adults and, consequently, worsen performance and increases in the number of artifacts in the EEG signal.

### EEG recording and preprocessing

A 32-channel EEG (Ag/Cl electrodes mounted in an elastic cap according to the 10/20 international system; Electro-Cap International, Inc., Eaton, OH, United States) was recorded during the performance of the sentence comprehension reading task using NeuroScan SynAmps (Compumedics NeuroScan) amplifiers. The left earlobe (A1) signal was used as an online reference, and the right earlobe (A2) was also recorded. Ocular movements were also recorded with two electrodes placed on the external canthus and the supraorbital ridge of the left eye. The EEG was digitalized at 500 Hz, and a bandwidth from 0.1 to 100 Hz was used. Electrode impedances were maintained below 2 kΩ.

EEG recordings were processed offline using Scan 4.5 software (Compumedics NeuroScan). The signal was referenced to the averaged A1-A2 activity, and eye movements were automatically corrected to remove blinks [54]. The continuous EEG recording was segmented in epochs between 200 ms prestimulus and 1000 ms poststimulus per subject and experimental condition (agreement/low WM load, agreement/high WM load, disagreement/low WM load, and disagreement/high WM load) and baseline corrected using the 200 ms prestimulus signal. Given that adjectives in Spanish have a postnominal position, the segmentation performed was triggered to the adjectives in each sentence. Segments with absolute voltages greater than +/- 50 μV and with ocular or muscular artifacts were rejected. Epoch files for each condition were separately imported into EEGLAB [55] for statistical analyses.

### Behavioral analysis

Statistical analysis of the behavioral data (percentage of correct responses with respect to the number of stimuli presented and reaction times) was performed by conducting nonparametric permutation tests (10,000 permutations) using personalized MATLAB scripts and the “statcond” function [55]. To obtain a normal distribution of the data, the percentages of correct responses were transformed using the function ARCSINE [square root (percentage/100)] [56]. Two different analyses for each behavioral measure (accuracy and reaction times) were separately carried out. First, the agreement effect was separately tested for each working memory load condition (high and low) by performing two-way ANOVA with Group (h-PA and l-PA) and Agreement (agreement and disagreement) as factors. Second, to analyze the effects of working memory load, two-way ANOVA was conducted using the behavioral data differences between disagreement and agreement conditions, taking Group (h-PA and l-PA) and WM load (high and low) as factors.

### ERP analysis

The time window analyzed in terms of amplitude was concurrent with the presentation of the adjective in both WM load conditions.

#### Amplitude

ERP statistical analysis of amplitude was performed by conducting nonparametric permutation tests using 10,000 permutations as implemented in the EEGLAB function “statcond” [55] over all electrodes (Fp1, Fp2, F3, F4, C3, C4, P3, P4, O1, O2, F7, F8, T3, T4, T5, T6, Cz, Fz, Pz, FCz, CPz, CP3, CP4, FC3, FC4, TP7, TP8, FPz, Oz, FT7, and FT8). False discovery rate (FDR) correction was used to adjust p-values for multiple comparisons.

According to previous EEG literature regarding gender-agreement research, three ERP components were analyzed: LAN (300–500 ms), P600a (500–700 ms), and P600b (800–1000 ms). Component latencies were selected by visual inspection of the difference waves (i.e., disagreement minus agreement) and according to those reported in previous studies [10, 41, 42]. In previous literature older adults showed changes in the topographical location of the components. Therefore, we decided to include all electrodes in the analyses [10, 46].

Two different analyses were independently conducted using the mean amplitude values for each ERP component, as follows: 1) to assess the agreement effect for each WM load condition, two two-way ANOVAs were separately performed with Group (h-PA and l-PA) and Agreement (agreement and disagreement) as factors, and 2) to analyze the effects of WM load, a two-way ANOVA was conducted using the difference waves (disagreement minus agreement) with Group (h-PA and l-PA) and WM load (high and low) as factors. FDR correction was applied for multiple comparisons.

All EEG and Behavioral data files are available from figshare.com repository(https://figshare.com/s/be4d6f89b3761c8ef372).

## Results

### Behavioral

**Fig 2:**
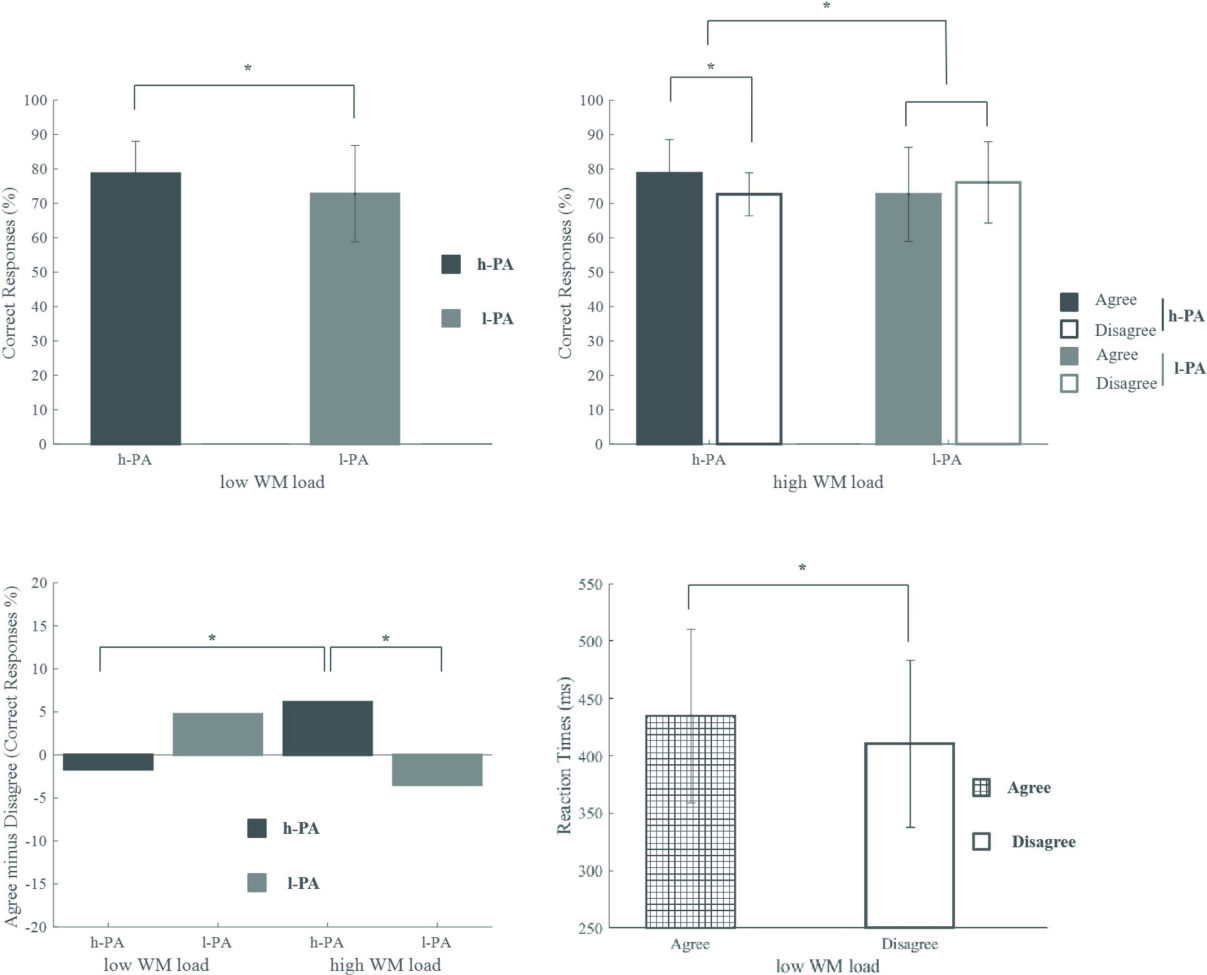
Behavioral results. Graphics of behavioral data with significant results are shown. A) Significant main effect of Group on accuracy during low WM load. Percentage of correct responses (agreement and disagreement conditions were merged) for h-PA individuals are shown in black and for l-PA individuals are shown in gray. B) Significant interaction of Group and Agreement on accuracy for the high WM load condition. The percentages of correct responses for each condition and group are displayed (h-PA data are shown in black and l-PA data are shown in gray). C) Significant interaction of Group by WM load on the effect of agreement for the percentage of correct responses. The percentage of the agreement effect for each WM load condition (low WM load, left, and high WM load, right) and group (h-PA data are shown in black and l-PA data are shown in gray) are depicted. D) Main effect of agreement on reaction times during low WM load. Reaction times (h-PA and l-PA groups are merged) for agreement (left) and disagreement (right) conditions are shown. h-PA = high physical activity group, l-PA = low physical activity group, WM = working memory. Error bars represent the standard deviation. Note that for a better illustration of the results, the percentage of correct responses is displayed instead of the ARCSINE values that were originally used for statistical analysis (see Method and behavioral results above).

Fig 2 graphically displays the significant results of the analysis of accuracy and reaction times reported below, in this section.

#### Percentage of correct responses

Regarding low WM load two-way ANOVA (Group by Agreement) results, a significant effect of Group (p = .04) was found, in which the responses of the h-PA group (mean = 78.54, standard deviation = 9.35) were more accurate than the responses of the l-PA group (mean = 72.72, standard deviation = 14.48), regardless of the agreement. No significant effect of Agreement (p = .61) or significant Group by Agreement interaction (p = .26) was found (Fig 2A).

Regarding high WM load two-way ANOVA (Group by Agreement) results, no significant effect of Group (p = .64) or Agreement (p = .50) was found. However, there was a significant Group by Agreement (p = .04) interaction. Pairwise comparisons revealed a significant effect of Agreement in the h-PA group (i.e., a higher percentage of correct responses in the agreement than in the disagreement condition: mean difference, MD = 6.33, p = .03) but not in the l-PA group moreover; groups significantly differed in the effect of Agreement (MD = 9.96, p = .04, see also the next analysis; Fig 2B).

With respect to the WM load effect (analyzing the agreement effect for accuracy, that is, the percentage of correct agreement responses minus the percentage of correct disagreement responses for each group and WM load condition) two-way ANOVA (Group by WM load) results, there was no significant effect of Group (p = .63) or WM load (p = .95). However, there was a significant Group by WM load interaction (p = .04). Within-subject post hoc analysis showed a significant difference in the effect of Agreement between high and low WM load conditions only in the h-PA group (MD = 8.08, p = .03). Importantly, the Agreement effect was positive for a high WM load, while for a low WM load, the effect was negative. Post hoc analysis of the comparison between groups revealed significant differences in the Agreement effect only for the high WM load condition with a positive effect of agreement in the h-PA group with respect to the l-PA group, which showed a negative effect (MD = 9.96, p = .04); this result corresponds to the significant interaction found in the previous analysis. No differences were observed between the high and low WM conditions for the l-PA group or between the groups in the low WM load condition (Fig 2C).

#### Reaction times

Low WM load two-way ANOVA (Group by Agreement) revealed a significant effect of Agreement (p = .008) with faster reaction times for the agreement than for the disagreement condition (MD = 24.10 ms) regardless of the group. No significant effect of Group (p = .50) or significant Group by Agreement interaction (p = .28) was found (Fig 2D).

High WM load two-way ANOVA (Group by Agreement) showed no significant effect of Group (p = .82) or Agreement (p = .09) and no significant Group by Agreement interaction (p = 0.80) was found.

WM load effect (analyzing the agreement effect for the reaction times, that is, the reaction times for the agreement condition minus the reaction time for the disagreement condition for each group and WM load condition) two-way ANOVA (Group by WM load) revealed there was no significant effect of Group (p = .44) or WM load (p = .50) nor a Group by WM load interaction (p = .44).

### ERP results

**Fig 3:**
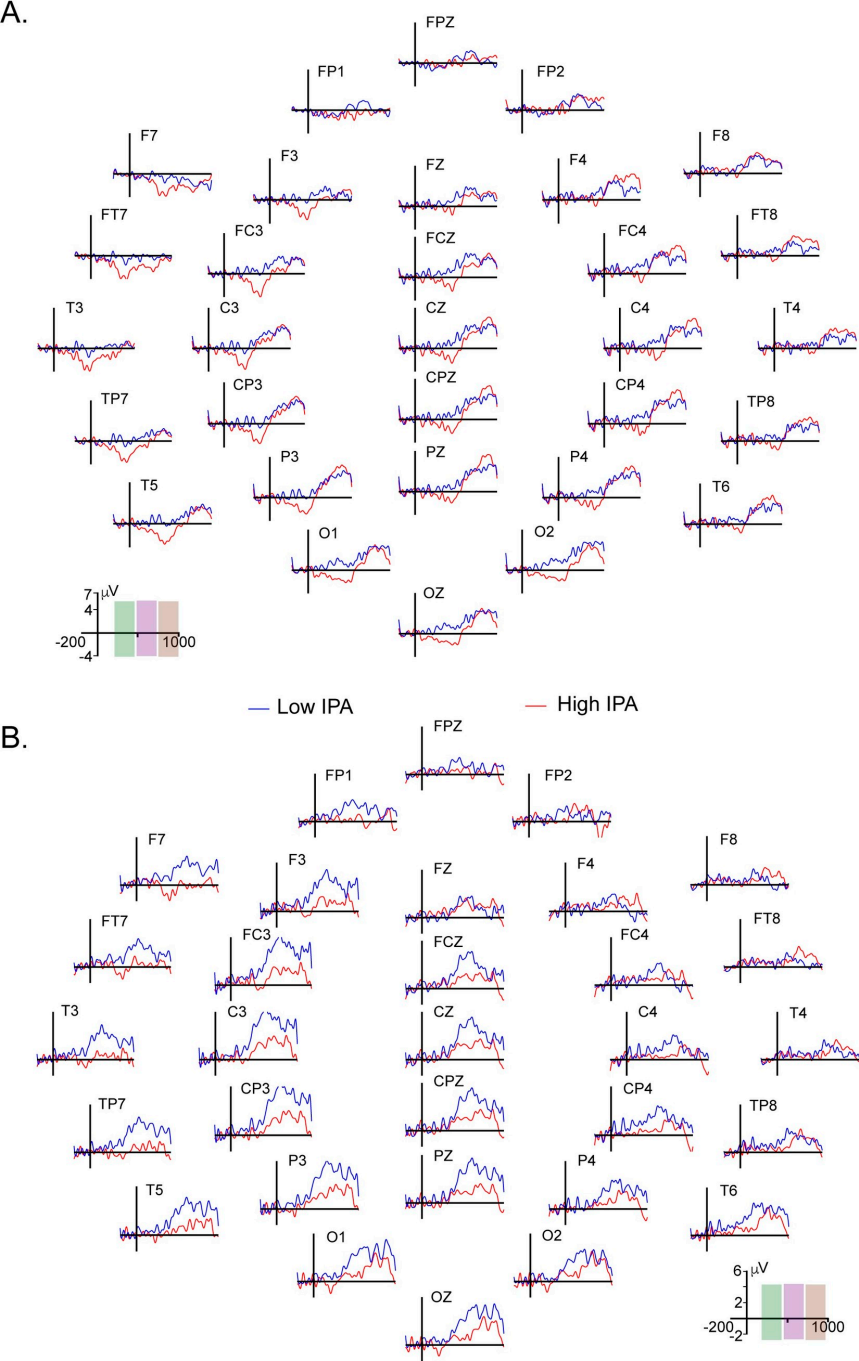
ERP waveforms of the effect of gender agreement during low and high working memory load conditions. Waveforms of the effect of gender agreement (the disagreement condition minus the agreement condition) for the (A) low WM and (B) high WM load conditions are shown. The low PA group is shown in blue lines, and the high PA group is shown in red lines. To illustrate the time windows used for each component analyzed LAN, P600a and P600b are shown in green, violet and brown shadows, respectively, on the electrode displaying the time-amplitude scale. Electrodes with significant p-values are displayed in Figs 4 and 5.

**Fig 4:**
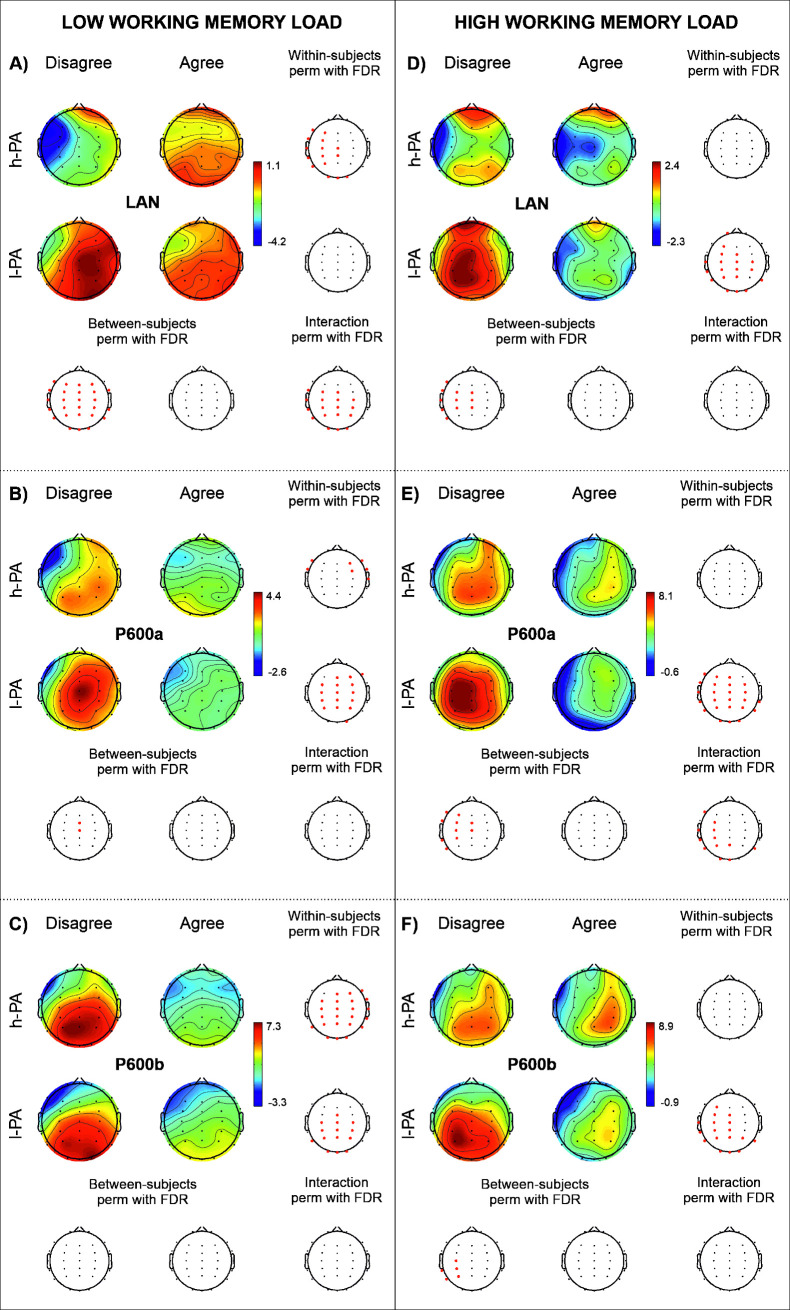
ERP results of the effect of gender agreement during low and high working memory load conditions. The results of the within- (first and second row of the third columns) and between- (first and second column of the third row) subject analyses and their interaction (lowest right head) are displayed for LAN (A and D), P600a (B and E) and P600b (D and F) components. Amplitude maps for disagreement (first column) and agreement (second column) conditions in the h-PA (high level of incidental physical activity; first row) and l-PA (low level of incidental physical activity; second row) groups are presented in each panel. Channels highlighted in red represent significant differences (p < .05) using nonparametric permutations with FDR correction for multiple comparisons (perm with FDR).

In Figs 3 and 4, ERPs of the agreement effect (disagreement minus agreement conditions) and the topographical distribution of the wave for each experimental condition are shown. LAN was observed at left anterior sites mainly in the low WM load condition, and its amplitude was greater for the h-PA group than for the l-PA group. In the high WM load condition, LAN was practically absent from the l-PA group. LAN was followed by two positive waves, P600a and P600b, over the central and centroparietal electrodes. In the low WM load condition, the P600a component was mainly observed over the right electrodes, while in the high WM load, this effect was observed over the left electrodes; the amplitude of P600a seemed to be greater in the high WM load condition for the l-PA group than for the h-PA group, while the P600b amplitude was greater in the low WM load condition for the h-PA group than for the l-PA group.

#### Amplitude effect of gender agreement

The effect of gender agreement was analyzed during low and high WM loads involving comparisons between agreement and disagreement conditions for each ERP component and WM load condition separately. The results are shown in Fig 4.

*a) Effect of gender agreement during low WM load*: *LAN*. Significant differences between agreement conditions were observed only in the h-PA group. The negative wave was significantly greater for the disagreement condition than for the agreement condition over the left fronto-centro-temporo-parietal electrodes and bilaterally in the occipital electrodes.

A significant effect of Group was found for the disagreement condition. There were significant differences between groups, where the h-PA group showed a greater negative amplitude over the left fronto-centro-temporo-parietal electrodes, while the l-PA group showed a greater positive wave amplitude over the right anterior, central and posterior electrodes.

A significant Agreement by Group interaction was observed over the central, parietal and occipital electrodes bilaterally but mainly over the left side. No additional differences were found (see Fig 4A).

*b) Effect of gender agreement during low WM load*: *P600a*. A significant effect of Agreement was found, with greater P600a component amplitudes in the disagreement condition than in the agreement condition in both groups; in the h-PA group, this effect was observed bilaterally over the fronto-temporal electrodes, whereas in the l-PA group, these differences were observed bilaterally over the fronto-centro-parietal electrodes.

A significant Group difference was found with greater amplitudes in the l-PA group over the Fz and FCz electrodes than in the h-PA group.

No significant interaction was observed (see Fig 4B).

*c) Effect of gender agreement during low WM load*: *P600b*. A significant effect of Agreement was found in which both groups (h-PA and l-PA) showed larger amplitudes of this positive wave for the disagreement condition than for the agreement condition. This effect was observed over the anterior, central and posterior electrodes in the h-PA group, while in the l-PA group, it was only observed over the central and posterior electrodes.

No significant differences between groups or in the Agreement by Group interaction were found (see Fig 4C).

*d) Effect of gender agreement during high WM load*: *LAN*. A significant difference between Agreement conditions was found where the l-PA group showed larger positive wave amplitudes for the disagreement condition than for the agreement condition over the anterior central and posterior electrode sites, mainly on the left side.

There were significant differences between groups for the disagreement condition; the h-PA group showed a greater amplitude of the LAN component than the l-PA group, whereas the positive wave observed in the l-PA group was greater than that observed in the h-PA group over the anterior, central and posterior electrode sites.

No significant interaction was found (see Fig 4D).

*e) Effect of gender agreement during high WM load*: *P600a*. Significant differences between agreement conditions were found, in which the l-PA group showed a significant widespread P600a effect (i.e., greater amplitudes for the disagreement condition than for the agreement condition).

Significant differences between groups showed that the l-PA group displayed greater amplitudes over the left electrode sites for the disagreement condition than the h-PA group.

A significant Agreement by Group interaction was observed over the anterior, central and posterior sites, mainly on the left side (see Fig 4E).

*f) Effect of gender agreement during high WM load*: *P600b*. Significant differences between agreement conditions were found, in which the l-PA group showed greater amplitudes for the disagreement condition than for the agreement condition over the left and midline fronto-centro-temporo-parietal electrode sites and bilaterally over the posterior sites; this effect was not observed in the h-PA group.

A significant difference between groups was observed with greater amplitudes for the disagreement condition in the l-PA than in the h-PA group over the left temporoparietal sites.

No significant Agreement by Group interaction was observed in the analysis of P600b (see Fig 4F).

#### Amplitude effect of WM load

**Fig 5:**
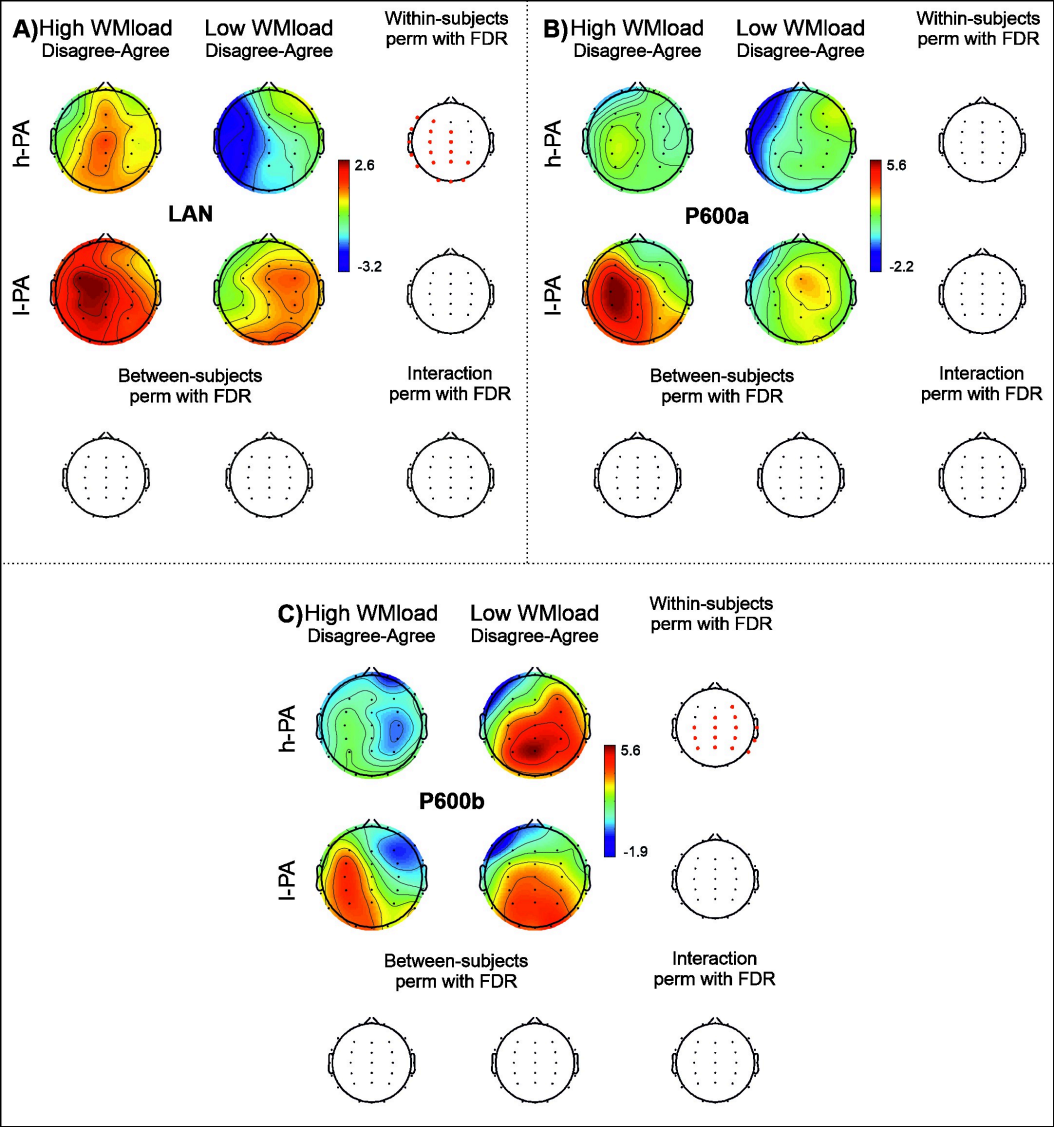
ERP results of the effect of WM load on gender-agreement detection. The results of the within- (first and second row of the third columns) and between- (first and second column of the third row) subject analyses and their interaction (lowest right head) are displayed for the LAN (left anterior negativity; A), P600a (B) and P600b (C) components. Amplitude maps for high working memory load (first column) and low working memory load (second column) conditions in the h-PA (high level of incidental physical activity; first row) and l-PA (low level of incidental physical activity; second row) groups are presented in each panel. Channels highlighted in red represent significant differences (p < .05) using nonparametric permutations with FDR correction for multiple comparisons (perm with FDR).

To evaluate the interaction between WM load and Agreement, comparisons between the high and low WM loads and between the h-PA and l-PA groups were separately conducted using the difference wave of the disagreement condition minus the agreement condition for each component. The results are shown in Fig 5.

*a) Effect of WM load*: *LAN*. Significant differences between WM load conditions were found in the h-PA group with a significantly greater LAN effect in the low WM load condition over the midline and left electrodes; this effect was not observed in the l-PA group.

No significant differences between groups or WM load by Group interaction were found (see Fig 5A).

*b) Effect of WM load*: *P600a*. No significant differences between WM load conditions or groups were found, nor were WM load by Group interaction found (see Fig 5B).

*c) Effect of WM load*: *P600b*. Significant differences between WM load conditions were found, in which the h-PA group showed a larger amplitude for the low WM load than for the high WM load condition over the anterior, central and posterior electrodes, mainly on the right side. No differences in the l-PA groups were found.

No additional differences between groups or WM load by Group interaction were found (see Fig 5C).

## Discussion

The findings of this study pointed out to a positive effect of incidental physical activity on morphosyntactic processing. Adults with h-PA seem to maintain more preserved the automatization of morphosyntactic processing, and more adaptative mechanisms to face the stimulus complexity compared to their peers with low l-PA.

### Behavioral evidence

We hypothesized that older adults with l-PA would show lower accuracy and longer RTs than older adults with h-PA in both WM load conditions [38, 40, 57]. Our results partially fit our expectations, as we observed that the group with h-PA displayed better accuracy than the older adults with l-PA during low WM load condition but not during high WM load condition (see Fig 2A). Our results suggest that different level of PA is associated with different morphosyntactic processing, regardless of the WM process; more specifically high levels of PA seem to assure better morphosyntactic processing than low levels of PA.

Although no differences in accuracy between groups were observed during high WM load condition, we found that the agreement effect (the disagreement condition minus the agreement condition) was significantly higher in the group with h-PA. This group showed better performance in the agreement condition than in the disagreement condition, while participants with l-PA did not display any significant differences between the experimental conditions (see Fig 2B). Moreover, in the group with h-PA, we observed an agreement effect during high but not low WM load conditions (see Fig 2C). In this respect, we suppose that high WM load condition entails more difficulties than those expected in low WM load condition, which become even greater when assessing the disagreement condition (i.e., the more complex condition in this case is the disagreement condition due to an additional computation of gender manipulation). Therefore, older adults with h-PA may trigger the expected mechanism in response to stimulus complexity [58]. This group may be focused on resolving the less complicated condition (i.e., agreement condition), and consequently, they displayed better performance in the agreement condition than the disagreement condition since, in the latter condition, an error repair is added to the reanalysis of the whole sentence. On the other hand, the group with l-PA performed in a similar way regardless of the level of difficulty of the task; moreover, in both WM load conditions, older adults with l-PA do not seem to respond as expected to the task’s complexity.

Regarding reaction times, our results did not confirm our hypothesis since no differences between groups were found. However, during low WM load condition, we found an effect of speed facilitation for the agreement trials in comparison with the disagreement trials; this effect was similar for both groups and disappeared when WM load increased (see Fig 2D). This unexpected result suggests that during morphosyntactic processing, the influence of different levels of PA on reaction times is less relevant than their effects on accuracy. In fact, some previous studies have demonstrated that morphosyntactic processing in healthy older adults tends to sacrifice response speed in favor of accuracy [10, 59].

Our behavioral results showed a different pattern of response in each group, which was modulated for the agreement and WM load only in h-PA individuals. This suggests not only that PA may influence morphosyntactic processing but also that the group with h-PA displays a behavioral response more associated with task complexity than participants with l-PA.

### Electrophysiological evidence

#### Agreement vs disagreement in low WM load condition

Previous studies [42] have reported the electrophysiological patterns associated with gender disagreement (a similar scenario to our low WM load condition), and these have described a greater amplitude in the disagreement condition than in the agreement condition in all components (i.e., LAN, P600a and P600b effects). In this study, our groups demonstrated significant differences in agreement effect, mainly at the first stage of processing (i.e., LAN effect); the group with h-lPA displayed a significant LAN effect, as previous studies have reported [42], whereas the other group displayed similar amplitude in both agreement conditions (i.e., no LAN effect). We presume that the group with l-PA probably uses fewer neural resources for disagreement condition processing than necessary to complete this stage (see Fig 4A). Differences between groups in the disagreement condition seem to support this idea because the group with l-PA showed a smaller LAN amplitude than that observed in the group with h-PA in this condition. Given that the LAN component has been associated with morphosyntactic processing [34, 36, 60], our findings may suggest an ineffective brain response for the l-PA group during this processing in the condition with more complexity (i.e., disagreement condition).

It has been stated that when there is no significant LAN effect, morphosyntactic processing may be computed in later stages [61], which would be reflected in the amplitude modulation of the following component, that is, the P600a component. Then, a greater P600a amplitude is expected in the conflicting condition. In this regard, our groups showed a significant P600a effect (i.e., greater amplitude in the disagreement condition than in the agreement condition) as prior studies have reported [42], but the group with l-PA displayed a more widespread P600a effect than the other group (see Fig 4B); moreover, differences between groups were observed only in the disagreement condition (i.e., the group with l-PA showed greater P600a amplitude than that observed in the group with h-PA). The P600a component has been associated with the integration of sentence information [41]; therefore, the greater P600a amplitude observed in the l-PA group during the disagreement condition may be a reflection of the integration of sentence information [41] and error repair of morphosyntactic processing [61] that was not executed in the previous stage of processing.

In the next processing stage, a generalized mapping of sentences may be performed [34, 41, 44]; this processing has been associated with the P600b component. In this study, both groups displayed a significant P600b effect, but no differences between groups were found. We presume that at this processing level, both groups have resolved any conflict in sentence processing (see Fig 4C).

The group with l-PA displayed unusual brain electrical activity (i.e., no LAN effect) respect to previous findings in healthy older adults. These subjects fail to display the normal automatic syntactic processing as reflected by the LAN effect in the first stage of processing and then, they must display greater cognitive effort in posterior stages as reflected by a more widespread in P600a effect. Therefore, we suppose that higher PA, regardless of the WM load, could positively influence morphosyntactic processing.

#### Agreement vs disagreement in high WM load condition

Prior studies have reported that subjects show ERP modulations due to stimulus complexity or that WM load is increased; in particular, smaller or no LAN effects [10, 38] and smaller P600a and P600b effects have been described [10, 38–40]. No LAN effect was observed in older adults with h-PA, which may reflect difficulties in processing the morphosyntactic features of sentences. These participants seem to incur a similar processing cost in the two agreement conditions (see Fig 4D). This fact suggests that a greater amount of neural resources than expected was spent in the agreement condition or lower than expected neural resources were displayed for the disagreement condition. In both scenarios, the subjects with h-PA would reflect difficulties in processing sentences in the agreement or disagreement condition. However, this electrophysiological pattern has been reported in healthy older adults without risk of cognitive decline when WM load increase, and has been associated with age-related changes [10].

On the other hand, participants with l-PA did not show this pattern. For instance, the LAN component was observed in the agreement condition. However, in the disagreement condition, positive values in the time window of LAN component were found, which may be a paradoxical LAN effect (see Fig 4D). We suppose that this positive wave may represent the P300b component. This component appears when the subject requires enhanced focus during stimulus detection relative to the contents of working memory [62]. In other words, the subjects with l-PA may analyze adjectives based on previous morphosyntactic errors saved in their working memory instead of automatically retrieving grammatical information that matches the actual word with the previous one.

The paradoxical effect in ERPs has been observed only in pathological populations [63, 64], but in our study, both groups were composed of healthy older adults. Hence, we suppose that the lack of LAN effect in the group with h-PA and the paradoxical LAN effect component at this latency in the group with l-PA might be a reflection of failures in the first processing stage mainly for the most complicated condition (i.e., disagreement). However, the failures were different for each group. The group with I-PA seems to show an unexpected electrophysiological pattern, which may involve problems in automatic grammatical processing.

At the next stage of processing, differences between groups were also found in the P600a and P600b effects. The group with h-PA did not show a significant P600 effect (i.e., P600a and P600b effects), whereas the l-PA group showed significant effects (see Fig 4E and 4F). Moreover, the differences between groups were observed only in the disagreement condition (i.e., the group with l-PA showed greater P600 amplitude than the group with h-PA). However, the reason the group with h-PA did not exhibit the P600 effect could be explain with the findings of Gunter et al. [57]: a smaller P600 amplitude is observed when the sentence is more complex or entails a greater WM load. We suppose that the group with h-PA seems to display a smaller amplitude in the P600 component in the disagreement condition than in the agreement condition, as the disagreement condition is the most complex condition, resulting in a similar amplitude in both agreement conditions for the h-PA group. In this regard, a smaller P600 amplitude may reflect a greater processing cost to integrate sentence information and to trigger the generalized mapping of the sentence.

On the other hand, the group with l-PA seemed to allocate a greater cost in the disagree condition; we suppose that these subjects most likely modified their allocation of neural resources in the P600 component to compensate for the difficulties to automatically retrieve grammatical information. This electrophysiological pattern has not been reported. We suggest that participants with l-PA seem to substitute morphosyntactic analysis by other processes during the P600 component. The morphosyntactic information may be recovered using the semantic information of the sentence [34, 61] or information about other aspects of the sentence.

Therefore, persons with h-PA showed an electrophysiological pattern more similar to that observed in healthy older adults without risk of cognitive decline [10] when WM load increased. Additionally, the group with l-PA seemed to allocate neural resources in a different way than expected.

#### High vs low WM load conditions

We expected to find differences between groups regarding the WM load effect, but we found differences only within the group with h-PA in two components (i.e., LAN and P600b effect). The group with h-PA displayed smaller LAN and P600b effects when WM load was increased, which was not observed in the other group (see Fig 5A and 5C); these patterns are expected in older adults without a risk of cognitive decline [10]. On the other hand, the group with l-PA did not display the expected WM load effect in any component. We suppose that they showed a different effect on ERP components because they had to face greater difficulties in disagreement processing in both WM load conditions than those observed in the other group.

Our groups did not show a WM load effect in the P600a component, and we assume that both groups had a similar cost of resources in low and high WM loads when they had to integrate sentence information.

Our results suggest that each group used different processing strategies to face the high WM load condition. The group with h-PA displayed the expected WM effect; the subjects seem to adapt to the task’s complexity, which was not observed in the other group. Given that the group with l-PA displayed problems to automatize the morphosyntactic processing, we suppose that in low WM load, this group spent greater neural resources reaching those generated by increases in the WM load; these will make no differences between WM load for this group.

### Overview

Even when the behavioral performance and electrophysiological results were not completely consistent with our hypothesis, the electrophysiological pattern in both groups matches with behavioral findings in low WM load condition. For the group with h-IPA the expected electrophysiological pattern seems to be associated with better performance and, most likely, with the compensation of the age-related effects. In contrast, for the other group, the non-expected brain electrical activity might be associated with a decline in the information processing that has not been completely compensated before the behavior is executed, negatively affecting the behavioral outcome.

However, in high WM load condition, both groups displayed a different electrophysiological pattern, resulting in similar behavioral performance. The group with h-PA displayed an electrophysiological pattern more similar to those observed in older adults without a risk of cognitive decline in both WM load conditions [10], while the group with l-PA seemed to trigger another electrophysiological pattern (i.e., a lack of LAN in low WM load condition; a paradoxical LAN effect in high WM load condition), which seems to imply a less adaptative manner of neural resources management mainly in early stages of sentence processing.

We also expected a greater effect of WM load in the l-PA group than in the other group, but the WM load effect was only observed for the h-PA group, not only in behavioral performance but also in their electrophysiological pattern. We suppose that the unexpected neural resources management (e.g., the P300b component appeared instead of a LAN) triggered by the I-PA group did not allow us to observe the WM load effect.

Hence, it seems that high levels of PA could exert a nonspecific effect on the brain and, as a consequence, positively affect all cognitive processes [65] involved in sentence processing, particularly in morphosyntactic processing in low WM load condition, and the use of the expected strategy when WM load increases.

According to the hypothesis of neural flexibility [66], individuals performing structured physical activity (e.g., athletes) have more adaptation capacity than those who do not practice structured physical activity; this could explain the strategy used by the participants with h-PA when WM load increased, i.e., higher neural flexibility. Perhaps individuals with h-PA utilized the expected strategy based on condition complexity, whereas the other group utilized a different strategy because they have to overcome their problems with morphosyntactic processing and additionally deal with the WM load increase.

In terms of the network architecture of the human brain, it has been proposed that the compartmentalization of functions improves brain responses against brain deterioration [58]. In fact, the capacity of each module to function and modify its operation without affecting other modules enables higher adaptability. In our study, the results obtained in the group performing h-PA could reflect a higher compartmentalization as a function of the WM load increase. Indeed, it is known that educational and lifestyle factors, such as the level of physical activity, enhance neural flexibility and adaptive advantages [58, 67]. Apparently, despite the adverse effects of aging on cognition, individuals who perform more physical activity can maintain better cognitive status. This statement has been confirmed by numerous studies using different types of exercise training, be it structured or unstructured.

### Limitations

Further studies are necessary to disentangle the mechanism related to the effect of incidental physical activity on WM and sentence processing. Among the limitations of our study, we know that in terms of PA assessment, it was evaluated by means of a self-report inventory, and objective measures were not obtained. However, the YPAS has shown high reliability and test-retest reliability and correlates with accelerometry measures [24, 68]. For instance, De Abajo et al. [24] found a significant correlation between vigorous and moving indexes with BMI and accelerometry measures. This finding supports our results in which the groups significantly differ in both vigorous index and BMI (we must be cautious with the results since this could be likely influenced by other factors not measured here, such as nutrition and heritability). The fact that objective measures were not obtained indicates that we cannot state causal relationships between variables; nevertheless, associations between incidental vigorous physical activity and cognitive processing were explored.

On the other hand, we have a small sample in our study due to our strict inclusion criteria (e.g., their performance and the minimal number of EEG segments required for the analysis). This fact may be a limitation; we suppose that differences between high and low PA groups could be more evident by recruiting a larger sample, even though we used the appropriate statistical procedures to answer our research questions and to solve the problem of sample size.
